# The Impact of COVID-19 on Carotid–Femoral Pulse Wave Velocity: A Systematic Review and Meta-Analysis

**DOI:** 10.3390/jcm12175747

**Published:** 2023-09-04

**Authors:** Iwona Jannasz, Michal Pruc, Mansur Rahnama-Hezavah, Tomasz Targowski, Robert Olszewski, Stepan Feduniw, Karolina Petryka, Lukasz Szarpak

**Affiliations:** 1 Department of Geriatrics, National Institute of Geriatrics, Rheumatology and Rehabilitation, 02-637 Warsaw, Poland; 2 Research Unit, Polish Society of Disaster Medicine, 05-806 Warsaw, Poland; 3 Department of Public Health, International Academy of Ecology and Medicine, 02-091 Kyiv, Ukraine; 4 Chair and Department of Oral Surgery, Medical University of Lublin, 20-093 Lublin, Poland; 5 Department of Gerontology, Public Health and Education, National Institute of Geriatrics Rheumatology and Rehabilitation, 02-637 Warsaw, Poland; 6 Department of Gynecology, University Hospital Zurich, 8091 Zurich, Switzerland; 7 Department of Obstetrics, University Hospital Zurich, 8091 Zurich, Switzerland; 8 Research Unit, Internal Medicine Clinic, 03-003 Warsaw, Poland; karolinapetryka2001@gmail.com; 9 Henry JN Taub Department of Emergency Medicine, Baylor College of Medicine, Houston, TX 77030, USA; 10 Institute of Outcomes Research, Maria Sklodowska-Curie Medical Academy in Warsaw, 00-136 Warsaw, Poland; 11 Research Unit, Maria Sklodowska-Curie Bialystok Oncology Center, 15-027 Bialystok, Poland

**Keywords:** COVID-19, SARS-CoV-2, pulse wave velocity, PWV, cfPWV, arterial stiffness

## Abstract

COVID-19 is a complex multisystemic disease that can result in long-term complications and, in severe cases, death. This study investigated the effect of COVID-19 on carotid–femoral pulse wave velocity (cfPWV) as a measurement to evaluate its impact on arterial stiffness and might help predict COVID-19-related cardiovascular (CV) complications. PubMed, Web of Science, Embase, and the Cochrane Library were searched for relevant studies, and meta-analysis was performed. The study protocol was registered in PROSPERO (nr. CRD42023434326). The Newcastle–Ottawa Quality Scale was used to evaluate the quality of the included studies. Nine studies reported cfPWV among COVID-19 patients and control groups. The pooled analysis showed that cfPWV in COVID-19 patients was 9.5 ± 3.7, compared to 8.2 ± 2.2 in control groups (MD = 1.32; 95% CI: 0.38–2.26; *p* = 0.006). A strong association between COVID-19 infection and increased cfPWV suggests a potential link between the virus and increased arterial stiffness. A marked increase in arterial stiffness, a known indicator of CV risk, clearly illustrates the cardiovascular implications of COVID-19 infection. However, further research is required to provide a clearer understanding of the connection between COVID-19 infection, arterial compliance, and subsequent CV events.

## 1. Introduction

The COVID-19 pandemic has had wide-ranging global effects, affecting a substantial proportion of the population worldwide. By June 2023, there were over 765 million confirmed cases, and almost 7 million people died as a result of the severe acute respiratory syndrome coronavirus 2 (SARS-CoV-2) [1,2]. Initially, there was limited information about COVID-19, and the disease was thought to be simply an acute respiratory condition. Since then, research has shown that COVID-19 is a complicated multisystemic disease that can lead to death or long-term complications after recovery [3,4]. One significant concern is the close link between COVID-19 and cardiovascular (CV) complications. Patients with pre-existing CV conditions are at a higher risk of an unfavorable prognosis for COVID-19 infection. Moreover, COVID-19 itself may directly or indirectly cause significant CV complications [5], which persist even after recovering from the virus [6,7]. Not only the severity of COVID-19 in the acute phase but also the duration of symptoms might have an effect on vascular function [8]. Long COVID-19 is described as a condition that can arise after recovery from the primary infection or an unresolved COVID-19 infection, which presents with ongoing symptoms that cannot be attributed to any other disease or condition. Using a conservative 10% estimate, at least 76 million people worldwide are affected by long COVID-19. Furthermore, studies suggest that 10–30% of non-hospitalized and 50–70% of hospitalized individuals experience long COVID-19 symptoms [9]. However, the actual numbers might be higher due to the vast number of unreported cases [10].

COVID-19′s cardiovascular (CV) manifestations include arrhythmias, asymptomatic myocardial damage, overt congestive heart failure, and thromboembolic events [5,11,12] and result from the virus’s direct cytotoxic effect or the subsequent systemic inflammatory cytokine storm. Endothelial dysfunction seems to be a crucial driver and mediator of the COVID-19 pathophysiologic pathways [13]. Vascular endothelial cells have the angiotensin-converting enzyme 2 cellular receptors (ACE2-R) and the transmembrane serine protease 2 (TMPRSS2), synergistically facilitating SARS-CoV-2 entry into host cells. Infected endothelial cells increase the production of cytokines, promoting inflammation and thrombosis [14]. The resulting vasculitis, which may affect different parts of the body, contributes to the multiorgan failure seen in some COVID-19 patients [15]. There is evidence suggesting that COVID-19 accelerates vascular aging on a macrovascular level [16]. Other proposed mechanisms contributing to cellular senescence and vascular stiffness include COVID-19-induced mitochondrial dysfunction, increased local formation of reactive oxygen species (ROS), and resulting oxidative telomere shortening [17]. Both endothelial dysfunction and continuous subintimal inflammation contribute to the rapid fragmentation of elastin fibers in the arterial wall and their substitution with stiff, fibrous tissue. Given that COVID-19-induced pulmonary fibrosis can only be reversed to a certain level, it has been proposed that arterial stiffness might be a long-term CV consequence in most patients, irrespective of the severity of the initial infection [15,18]. Notably, vascular changes, especially endothelial function and arterial stiffness, may last for a long time after the COVID-19 infection [19].

Arterial stiffness may serve as a reliable indicator reflecting the vascular system’s age and the comprehensive health of the CV system. It is an integrated biomarker that evaluates the cumulative detrimental effect on the arteries of genetic and environmental exposures, as well as the influence of established CV risk factors [20]. Numerous studies have established the correlations between arterial stiffness, as measured by pulse wave velocity (PWV), and the elevated risk of CV disease [21]. This correlation is independent of other traditional risk variables that are often considered to be risk factors [22,23]. In order to confirm the arterial stiffness in COVID-19 patients, other tests such as the augmentation index (Aix), the cardio-ankle vascular index (CAVI), the arterial stiffness index (ASI), Young’s modulus of elasticity, and pulse pressure (PP) were performed [24,25,26,27].

PWV is essential in assessing vascular age and may have a stronger correlation with CV disease onset than metrical age [28]. PWV is a technique that is noninvasive and reproducible, and carotid–femoral pulse wave velocity (cfPWV) is now regarded as the gold standard in assessing arterial stiffness. The progressive stiffening of the arteries adversely affects arterial–ventricular interactions, decreasing the vessel’s capacity to alter volume in response to changes in blood pressure, which in turn might lead to heart failure. CfPWV has a high prognostic value since it may help identify individuals who are at a higher risk not just for future CV events, but also for all-cause mortality [19,29].

The purpose of this research is to investigate the effect of COVID-19 on carotid–femoral pulse wave velocity (cfPWV) as a measurement of the complications of COVID-19 on arterial stiffness and subsequent CV complications.

## 2. Materials and Methods

### 2.1. Study Design

This study is a systematic review and meta-analysis conducted in adherence to the Preferred Reporting Items for Systematic Reviews and Meta-Analyses (PRISMA) standards [30] (Table S1). The research protocol was pre-approved by all co-authors registered in the PROSPERO registry (International Prospective Registry of Systematic Reviews) under registration number CRD42023434326.

### 2.2. Search Strategy

Two independent reviewers (I.J. and M.P.) evaluated potential papers. Discrepancies were resolved via further discussion or arbitration by a third reviewer (L.S.). The literature search covered the period between January 2020 and June 2023, covering the following databases: PubMed, Web of Science, Embase, the Cochrane Library, as well as Google Scholar. The search included the combination of keywords: “pulse wave velocity” OR “PWV” OR “arterial stiffness” AND “COVID-19” OR “SARS-CoV-2” OR “severe acute respiratory syndrome coronavirus-2”. Citations of listed studies were examined for further relevant literature. Only the most recent and comprehensive articles from identical authors were included to avoid duplicates. Furthermore, reference lists of relevant publications and systematic reviews were reviewed for potential inclusions. All references were consolidated in Endnote (version X9), duplicated entries were removed, and finally, Rayyan, a software screening tool, was used.

### 2.3. Inclusion and Exclusion Criteria

Studies qualified if they met the following inclusion criteria: research comparing cfPWV in patients with current or previous COVID-19 infection to a control group, as cfPWV is now regarded as the gold standard in assessing arterial stiffness. This method has a high prognostic value since it may help identify individuals who are at a higher risk not just for future outcomes as motioned in the introduction [19,29]. We excluded studies not detailing desired outcomes, other than cfPWV measurement of arterial stiffness, studies with unclear descriptions of COVID-19 infection, and studies that did not include a comparable group, non-English publications, and other types of publications such as the following: editorials, conference papers, reviews, and letters to the editor. In assessed studies, the study group was people who had been diagnosed with COVID-19 and had recovered. The control group was patients who had never had a positive COVID-19 test.

### 2.4. Data Extraction and Quality Assessment

Using a pre-defined data extraction form that was designed by L.S., the two independent reviewers (I.J. and M.P.) extracted the data from the research, and disagreements were mediated by the third reviewer (L.S.). The following information was extracted from the relevant publications: study characteristics (including first author, publication year, country of origin, study design, and research groups), and patient data (participant count, age, and carotid–femoral pulse wave velocity across groups). The Newcastle–Ottawa Quality Scale (NOS) was used in order to evaluate the level of methodological rigor that was present in each of the studies that were included in the analysis. Based on the selection, comparability, and exposure criteria, NOS allocates a potential four, two, and three stars, respectively. Studies achieving a NOS score ≥ 7 were deemed high quality [31].

### 2.5. Statistical Analysis

Statistical analyses used Review Manager (version 5.4, Nordic Cochrane Centre, Cochrane Collaboration, Odense, Denmark) and Stata (version 14, StataCorp, College Station, TX, USA) were used. The odds ratios (ORs) with 95% confidence intervals (CIs) were employed for dichotomous data, whereas mean differences (MDs) with 95% CIs were used for continuous data. Every statistical test was conducted using a two-sided approach, with a significance threshold of *p* < 0.05. For continuous outcomes presented as median, range, and interquartile range, the means and standard deviations were estimated using the methodology delineated by Hozo et al. [32]. The I^2^ statistic was used to determine the degree of heterogeneity, with values of 25% indicating low heterogeneity, values of 25–50% indicating moderate heterogeneity, and values more than 50% showing high heterogeneity [33]. If I^2^ was greater than 50%, a fixed-effects model was employed; otherwise, a random-effects model was used. Potential publication bias in the included studies was assessed via Egger’s test and funnel plots.

## 3. Results

### 3.1. Study Selection and Characteristics

**Figure 1:**
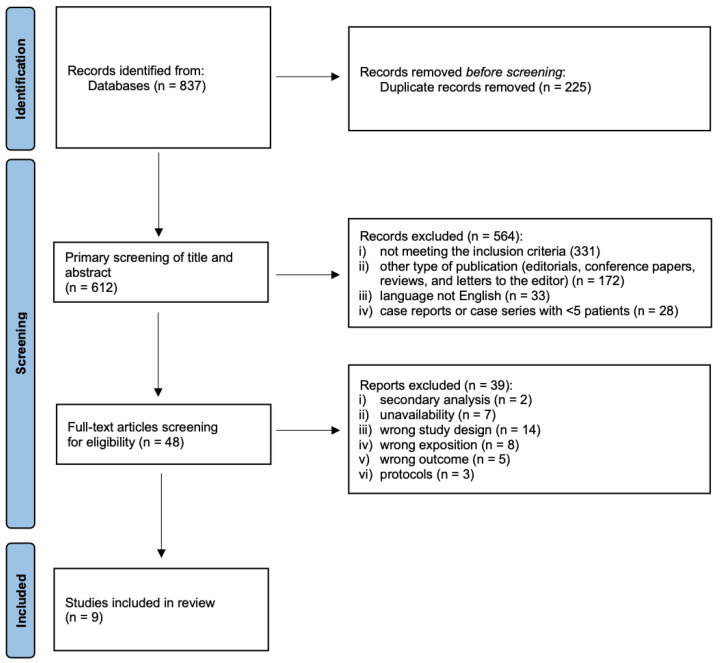
PRISMA systematic review flow diagram.

The bibliographic search results and selection process are shown in the PRISMA flow diagram (Figure 1). We identified 837 initial records, which were reduced to 612 after the elimination of duplicates. Titles and abstracts were screened, leading to the exclusion of 564 records. After assessing the remaining 48 articles for eligibility, we excluded 39 articles. As a result, nine studies were selected for qualitative synthesis and meta-analysis [34,35,36,37,38,39,40,41,42].

The essential characteristics of the included studies are outlined in Table 1. A total of nine studies that involved 536 patients were included in this meta-analysis. The mean age of the COVID-19 patient cohort was 50.8 ± 15.1 years, as compared to 51.3 ± 15.0 years in the control groups. Geographically, three studies were conducted in the United States, two in Greece, and the rest in Brazil, Austria, Romania, and the Netherlands. The sample size varied and ranged from 23 to 140 patients. Notably, the NOS scores of all the included studies were ≥7.

### 3.2. Meta-Analysis

**Figure 2:**
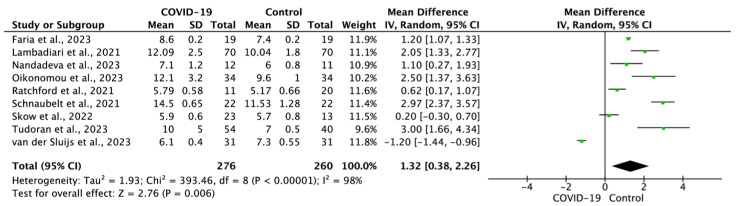
Forest plot of cfPWV in COVID-19 patients vs. non-COVID-19 controls [34,35,36,37,38,39,40,41,42]. The mean differences for individual studies are represented by the central point of each square, and the associated horizontal line indicates a 95% confidence range. The diamond shapes indicate the consolidated results.

All nine studies provided data on cfPWV values among COVID-19 patients and their respective control groups. The pooled analysis showed that cfPWV in COVID-19 patients was 9.5 ± 3.7, compared to 8.2 ± 2.2 in the control groups (MD = 1.32; 95% CI: 0.38 to 2.26; *p* = 0.006; Figure 2). It is important to note that the results from the sensitivity analysis did not alter the direction of the initial findings.

## 4. Discussion

Our meta-analysis revealed a significant correlation between COVID-19 infection and an increase in cfPWV [34,35,36,37,38,39,40,41,42]. However, it is worth noting that Van der Sluijs et al. did not observe such a correlation of cfPWV in their research [38], while Skow et al. found a positive, yet insignificant correlation [39]. Nevertheless, the remaining seven analyzed studies demonstrated a clear correlation between cfPWV and COVID-19 infection [34,35,36,37,40,41,42]. These findings suggest that COVID-19 may be responsible for the observed rise in arterial stiffness, which is a well-known marker of cardiovascular (CV) risk [43]. Arterial stiffness reflects changes in blood pressure, flow, as well as vascular diameter, and serves as an indicator of both the mechanical and functional properties of arterial walls. While the degradation of elastic fibers is the primary factor influencing arterial stiffness, other factors, such as fibrosis on replacement, collagen, elastin cross-linking, and medial calcifications also play important roles.

Studies by Townsend et al. and Lambadiari et al. highlighted that multiple factors contribute to arterial stiffness, including endothelial dysfunction, inflammation, oxidative stress, the turnover of extracellular matrix, and the regulation of smooth muscle tone in muscular arteries [40,44]. SARS-CoV-2 virus targets endothelial cells, entering the cell as soon as it binds to ACE2 receptors, decreasing the number of ACE2 receptors on the cell surface, leading to endothelial cell dysfunction [15,45]. The decreased endothelial function observed in COVID-19 patients results from viral infiltration and increased systemic inflammatory responses [46]. Cytokine storm targets specific receptors located on the surface of endothelial cells, leading to the activation of a number of different mediators, resulting in the activation of platelets and the release of leukocytes into circulation [47]. Uncontrolled systemic inflammation may directly stimulate arterial remodeling or cause adrenoceptor hyporeactivity, impairing vascular responsiveness. Additionally, nitric oxide (NO) deficiency in COVID-19 patients can exacerbate endothelial dysfunction and lead to increased arterial stiffness, impaired smooth muscle relaxation, and increased oxidative stress (further exacerbated by the cytokine storm) [48]. Changes in NO bioavailability, combined with SARS-CoV-2’s direct action on endothelial cells after binding to ACE2 receptors, can influence the functions of vascular smooth muscle cells and induce structural alterations in the vascular wall’s extracellular matrix, promoting arterial stiffness [49].

This arterial stiffness raises the risk of CV complications, including high blood pressure, heart attacks, and strokes, exerting additional strain on the heart. People with pre-existing CV conditions are particularly susceptible. A study by Faria et al. showed that COVID-19 patients, compared to their healthy counterparts, experienced over-activation of the sympathetic nervous system, vascular dysfunction, decreased physical fitness, and elevated cfPWV values (higher by 1.12 m per second) [34]. This is concerning, considering that previously published studies established PWV as a strong predictor of future CV events and all-cause mortality, and showed that the predictive power of arterial stiffness is higher in subjects with a higher baseline CV risk. This in turn suggests that an increase in arterial stiffness contributes to the elevated CV risk observed in COVID-19 survivors [29]. Elevated risks of stroke are consequences of both COVID-19 and increased arterial stiffness [50]. It is concerning that the effects of COVID-19 seem to last beyond the acute phase of the disease, as the virus may induce post-acute sequelae from COVID-19 (PASC). Nandadev et al. highlighted heightened arterial pressure and cfPWV values in PASC patients, suggesting they could develop CV problems at a faster rate [36]. It is interesting to note that a meta-analysis by Menezes et al. demonstrated that CV disease in COVID-19 patients had both cardioembolic and cryptogenic etiology [51], while factors like atherosclerosis were not directly linked to a COVID-19 positive result. Atrial fibrillation, coronary artery disease, diabetes, and hypertension were shown to be the most prevalent risk factors among COVID-19-positive individuals, increasing the risk of CV disease [51,52].

Ratchford et al. demonstrated a strong association between increased cfPWV and mortality among COVID-19 patients, particularly those with pre-existing chronic conditions, including CV disease [41]. Furthermore, a study by Schnaubelt et al. found that cfPWV among COVID-19 patients who survived the disease was significantly lower than in healthy patients, indicating a potential link with long-term complications [42]. Additionally, Kumar et al. showed increased cfPWV in severe COVID-19 cases as compared to non-severe cases [53]. We can assume that COVID-19 influences arterial stiffness, and this effect correlates with the severity of symptoms.

Research also shows that pre-existing conditions are also major factors that can accelerate arterial aging in the course of COVID-19. Tudoran et al. demonstrated a correlation between aortic and arterial stiffness, as well as diastolic dysfunction, in seemingly healthy individuals with post-acute COVID-19 syndrome patients. Their findings showed that women with a history of PASC and metabolic syndrome showed elevated cfPWV values and metrics of worsening of their diastolic dysfunction [35]. Throughout a six-month observation period, the values showed improvement; however, they did not revert fully. Oikonomou et al. evaluated cfPWV as well as the impairment of the left ventricle function measured by global longitudinal strain in the 6-month observation. While improvement was noted in both parameters, the values are still worse than in the control group, which may support the hypothesis that after recovering from COVID-19, and there is an increase in both arterial stiffness and the risk of adverse CV events in comparison to the general population [37]. Similarly, a 12-month follow-up study by Iconomidis and their team found COVID-19 survivors to still possess higher cfPWV values compared to controls at the 12-month follow-up evaluation. The authors showed considerable improvements in oxidative stress (levels of MDA), CFR, and myocardial work measures, in addition to a borderline improvement in left ventricular strain, which, nevertheless, continued to be impaired in comparison to the controls [54].

Another crucial area of research pertains to the management of post-COVID complications, including chronic arterial stiffness. While arterial stiffness is not easily reversible with medication or surgical interventions, additional therapies can be explored. One potential approach is the implementation of post-COVID-19 rehabilitation, which could help alleviate symptoms and improve overall outcomes. Comprehensive rehabilitation strategies, including exercise, physiotherapy, lifestyle changes, and cardiovascular rehabilitation, might help combat the long-term implications of arterial stiffness and increase the patient’s quality of life post-COVID. Gounaridi et al.’s research showed that a three-month cardiopulmonary post-acute COVID-19 rehabilitation significantly improved PWV, reducing it from 8.2 ± 1.3 m/s to 6.6 ± 1.0 m/s. Thus, rehabilitation could facilitate the recovery of endothelium-dependent vasodilation and arteriosclerosis [55]. Furthermore, exercise training conducted at home lowered cfPWV by a mean of −2.0 ± 0.6 m/s and has the potential to be an invaluable supplement to post-COVID rehabilitation [56].

However, it is essential to consider that the effect of COVID-19 may be dependent on the mutation of the virus. Skow et al. conducted their research on individuals during the Omicron wave of infections and found that arterial stiffness did not differ significantly between groups of individuals who had the Omicron variant of COVID-19 and controls who had never been exposed to COVID-19. According to these findings, the Omicron variant does not pose a threat to the CV health of young, vaccinated individuals who are otherwise healthy [39]. Nevertheless, there are no studies assessing other subtypes and mutations of COVID-19. Therefore, the correlation between the specific COVID-19 types and arterial stiffens remains inconclusive. Future research could provide us with more precise insights.

The measurement of cfPWV has significant clinical implications in terms of risk assessment and timely medical interventions to prevent COVID-19-related mortality and CV complications, particularly in hospitalized patients, patients with a severe disease course, or those who simply struggled with COVID-19. The timely identification of patients with increased arterial stiffness allows for the implementation of appropriate early medical interventions, including aggressive blood pressure management, optimization of medication regimens, and lifestyle changes, such as dietary changes, regular exercise, and smoking cessation. By implementing these interventions early on, healthcare providers can potentially mitigate the adverse CV effects of COVID-19 and improve patient outcomes. Moreover, longitudinal cfPWV monitoring in hospitalized COVID-19 patients can provide valuable information on the progression of arterial stiffness over time, enabling healthcare professionals to implement personalized treatment strategies, make necessary adjustments, and evaluate the effectiveness of interventions. By closely monitoring cfPWV, clinicians can track the response to treatment, identify any worsening of arterial stiffness, and promptly modify the management plan accordingly.

While our study brings valuable insights, it is essential to acknowledge its limitations. To the best of our knowledge, this is the first meta-analysis examining the influence of COVID-19 disease on arterial stiffness evaluated by cfPWV. The available research on studies investigating the connection between cfPWV and COVID-19 remains limited, both in terms of the number of studies and participant numbers. Furthermore, the observation window is short, covering the period between 2021 and 2023.

## 5. Conclusions

There is a strong association between COVID-19 infection and an elevated cfPWV, indicating a potential link between the virus and increased arterial stiffness. The substantial rise in arterial stiffness, an established indicator of CV risk, clearly shows the profound impact of COVID-19 on both immediate and long-term health outcomes. By accurately identifying individuals with augmented arterial stiffness, clinicians can tailor interventions and implement strategies that are more targeted toward lowering the CV risks associated with COVID-19. This will also facilitate timely medical and rehabilitation interventions for patients. However, further research is required in order to provide a clearer understanding of the connection between COVID-19 infection, arterial stiffness, and subsequent CV events. Thus, cfPWV measurements will be more useful as a diagnostic and prognostic instrument.

## Figures and Tables

**Table 1:** Characteristics of included studies.

Study	Country	Study Group	No. of Patients	Age	Sex, Male	NOS Scale
Faria et al., 2023 [34]	Brazil	COVID-19	19	47 ± 8	12 (63.2%)	8
		Control	19	43 ± 10	11 (57.9%)	
Tudoran et al., 2023 [35]	Romania	COVID-19	54	47.76 ± 5.43	NS	7
		Control	40	49.47 ± 5.14	NS	
Nandadeva et al., 2023 [36]	United States	COVID-19	12	48 ± 9	NS	7
		Control	11	50 ± 13	NS	
Oikonomou et al., 2023 [37]	Greece	COVID-19	34	57.2 ± 12.9	26 (76.5%)	8
		Control	34	57.4 ± 12.8	23 (67.6%)	
Van der Sluijs et al., 2023 [38]	The Netherlands	COVID-19	31	57.5 ± 3.0	17 (54.8%)	7
		Control	31	56.5 ± 3.0	17 (54.8%)	
Skow et al., 2022 [39]	United States	COVID-19	23	23 ± 3	9 (39.1%)	8
		Control	13	26 ± 4	6 (46.2%)	
Lambadiari et al., 2021 [40]	Greece	COVID-19	70	54.53 ± 9.07	44 (62.85%)	9
		Control	70	54.77 ± 8.95	44 (62.85%)	
		Control	34	57.4 ± 12.8	23 (67.6%)	
Ratchford et al., 2021 [41]	United States	COVID-19	11	20.1 ± 1.1	NS	9
		Control	20	23.0 ± 1.3	NS	
Schnaubelt et al., 2021 [42]	Austria	COVID-19	22	76.0 ± 4.25	11 (50.0%)	8
		Control	22	75.8 ± 4.0	10 (45.5%)	

## Data Availability

The data that support the findings of this study are available on request from the corresponding author (L.S.).

## Supplementary Materials

The following supporting information can be downloaded at the following link: https://www.mdpi.com/article/10.3390/jcm12175747/s1. Table S1: PRISMA 2020 Checklist.
